# Crosstalk between Brassinosteroids and Ethylene during Plant Growth and under Abiotic Stress Conditions

**DOI:** 10.3390/ijms19103283

**Published:** 2018-10-22

**Authors:** Petra Jiroutova, Jana Oklestkova, Miroslav Strnad

**Affiliations:** Laboratory of Growth Regulators, Centre of the Region Haná for Biotechnological and Agricultural Research, Institute of Experimental Botany ASCR & Palacký University, Šlechtitelů 27, 78371 Olomouc, Czech Republic; pe.jiroutova@gmail.com (P.J.); miroslav.strnad@upol.cz (M.S.)

**Keywords:** brassinosteroid, ethylene, plant growth, stress tolerance

## Abstract

Plant hormones through signaling networks mutually regulate several signaling and metabolic systems essential for both plant development and plant responses to different environmental stresses. Extensive research has enabled the main effects of all known phytohormones classes to be identified. Therefore, it is now possible to investigate the interesting topic of plant hormonal crosstalk more fully. In this review, we focus on the role of brassinosteroids and ethylene during plant growth and development especially flowering, ripening of fruits, apical hook development, and root and shoot growth. As well as it summarizes their interaction during various abiotic stress conditions.

## 1. Introduction

To date, nine groups of plant hormones have been identified, i.e., auxins, brassinosteroids, cytokinins, gibberellins, ethylene, jasmonic acid, strigolactones, abscisic acid, and salicylic acid. Genetic and physiological studies have revealed the critical roles of these phytohormones in plant growth and development, as well as plant responses to various biotic and abiotic stresses [1].

Brassinosteroids (BRs) are a class of polyhydroxylated steroidal hormones that regulate various aspects of plant growth and development. They were initially identified based on their growth promoting activities, but subsequent physiological and biochemical studies have revealed additional functions of BRs in regulating a wide range of processes, including seed germination, senescence, polarization of cell membranes and photosynthetic efficiency. Recently, it has been reported that BRs increase plant tolerance to stress factors, e.g., salt, drought, temperature, and heavy metals [2,3].

Ethylene, the first identified gaseous plant hormone, has a simple two-carbon structure. Nevertheless, it has been shown to regulate many diverse developmental and physiological processes in plants. In etiolated seedlings, ethylene causes a typical “triple response”, consisting of exaggerated curvature of the apical hook, inhibition of stem elongation and radial swelling of the hypocotyl. Besides the triple response, ethylene is involved in every phase of the plant life cycle, e.g., seed germination, root hair development, root nodulation, flower senescence, abscission, and ripening of fruit. Moreover, ethylene acts as a stress hormone during biotic and abiotic stress conditions [4].

**Figure 1:**
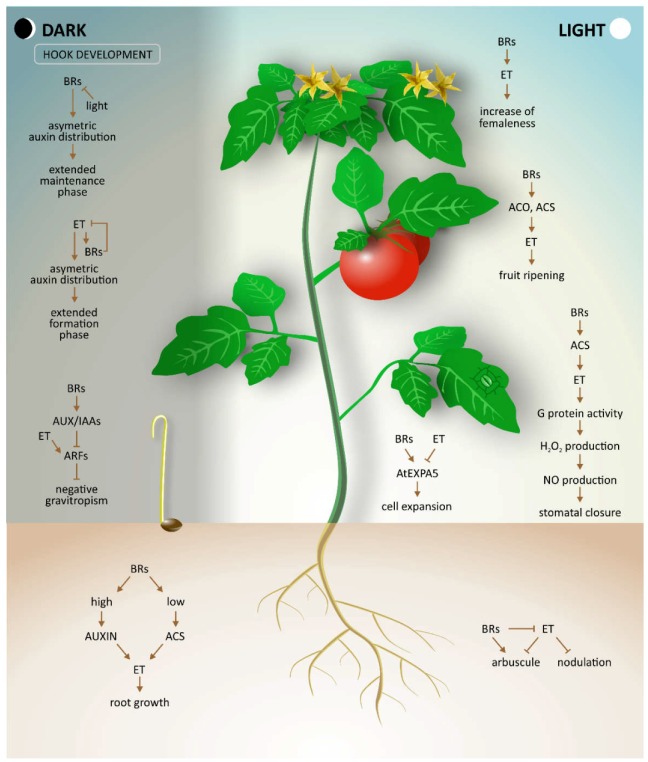
Simplified model of brassinosteroids and ethylene crosstalk showing the effects of these two hormones during plant growth and development. Arrows indicate stimulatory effect and blunted lines indicate inhibitory effect. See corresponding sections of the text for details and references.

Plant hormone crosstalk is a complex topic of broad and current interest. In this review, we provide a comprehensive overview of the interaction of BRs and ethylene during plant development and under abiotic stress conditions (Figure 1).

## 2. Root Growth

Roots are an important underground part of vascular plants with two main functions—fixing plants in a soil and absorption of water and nutrients. Hence, well-developed roots are crucial for proper growth and development of the whole plant. In higher plants, control of root growth is mainly associated with auxins and cytokinins as positive and negative regulators, respectively [5]. However, other plant hormones and their interactions play an important role in diverse growth processes in roots. In addition, other signal molecules, such as reactive oxygen species (ROS), play valuable roles in root development [6].

Interaction between BRs, ethylene, and ROS has been examined by Lv et al., 2018 [7]. In their study, an *Arabidopsis* mutant (*det2-9*) with a defect in BR synthesis was identified based on its short-root phenotype by EMS mutant screening. Because both ROS and ethylene signaling were enhanced in the *det2-9* mutant, it was suggested that the short-root phenotype resulted from hyper-accumulation of ethylene and superoxide anions (O_2_^−^). Exogenous application of BRs showed that they either positively or negatively regulated the biosynthesis of ethylene depending on the applied concentration. In seedlings treated with a low concentration (10 or 100 nM) of 24-epibrassinolide (EBL), ethylene production was greatly reduced, whereas treatment with higher concentrations of EBL (≥ 500 nM) caused a strong increase. Accordingly, BRs at low concentrations (10–100 nM) inhibited expression of ethylene response factors (ERFs), whereas high concentrations (≥500 nM) enhanced ERF expression, consistent with the observed changes in ethylene levels after treatment with BRs. Chromatin immunoprecipitation (ChIP)/qPCR analysis confirmed direct interaction of ACSs (1-aminocyclopropane-1-carboxylic acid synthases, crucial enzymes in the ethylene biosynthetic pathway) by BES1 or BZR1 (brassinosteroid-regulated transcription factors). This interaction appeared as inhibition because over-expression of both BES1 and BZR1 strongly suppressed the activity of ACS promoters. qRT-PCR results using BR-insensitive mutants indicated increased expression of ACSs. Altogether, these findings suggest that at physiological levels, BRs regulate the repression of ethylene biosynthesis via the BES1 and BZR1 transcription factors, whereas at high levels, BRs induce ethylene biosynthesis by increasing the stability of ACSs and influencing auxin signaling, increasing ethylene production. It was also shown that BRs (via the peroxidase pathway) inhibited the synthesis of O_2_^−^, thereby controlling root growth, because of hyper-accumulation of O_2_^−^ contributed to the short-root phenotype in the *det2-9* mutant [7].

Not only is the regulation of longitudinal growth, but also directional growth, important for proper root development. Gravitropism and the elongation of roots can be modulated by various environmental signals. Singh and co-workers [8] showed that enrichment of the medium with glucose (Glc) broadly modulates seedling root growth direction and simultaneous application of BRs dramatically enhances this modulation. In particular, Glc caused root deviation from straight vertical growth and this deviation was dose-dependent on Glc content in the medium. Experiments suggested that Glc may enhance BR signaling via enhancing BRI1 endocytosis from the plasma membrane to early endosomes. Follow-up work [9] focused on the interplay of other phytohormones and Glc in controlling root directional growth. The main findings of this work were that the presence of cytokinins and ethylene could abolish deviation of roots growing on medium enriched with Glc/BRs and they (cytokinins and ethylene) could also act antagonistically with BRs in the case of directional growth regulation. Further experiments with various mutants suggested that cytokinin signaling works downstream to BRs and antagonizes the Glc induced root directional response via ethylene-mediated machinery [9].

## 3. Shoot Growth and Apical Hook Development

The growth of shoots is the direct result of cell elongation, which is controlled by a complex system of phytohormone interaction. BRs are plant hormones with strong cell-promoting activity. In 2014, Bergoci et al. [10] described one of many mechanisms by which BRs promote cell elongation. Their proposed model scheme included interference between BRs and a rapid alkalization factor (RALF) comprising peptides belonging to compounds with inhibitory activity on growth. Simultaneous treatment with AtRALF1 and brassinolide (BL) induced lower levels of AtRALF1-inducible cell wall remodeling genes *AtPRP1*, *AtPRP3*, and *AtHRGP2*, which are responsible for cell wall hardening and inhibition of further elongation. In additional experiments, it was observed that plants with a partially silenced *AtRALF1* gene showed increased levels of the expansine gene *AtEXPA5* involved in cell expansion [10]. A previous study [11] showed that exogenously applied BRs increase levels of AtEXPA5, suggesting an antagonistic effect between AtRALF1 and BR in the regulation of expansine genes. In contrast, ethylene was found to reduce AtEXPA5 expression levels, thereby regulating growth of the hypocotyl [12]. These results suggest that AtRALF1 and ethylene may act together to achieve the same effect [10].

Another study dealing with the influence of ethylene and BRs on hypocotyl development was published in 2013 [13]. The study involved screening and identifying mutant plants of *Arabidopsis* with an altered response to acsinone7303, which is a small molecule that can act like an uncompetitive inhibitor of ACS. Treatment of etiolated *eto1* mutant seedlings with acsinone reduced ethylene levels and suppressed the triple response. Several *ret* mutants with reduced sensitivity to acsinone7303 were identified and two of them (*ret8* and *ret41*) were characterized. Map-based cloning revealed that *ret8* carried a mutation in CESA6 (cellulose synthase 6, part of the primary wall CESA complex), whereas *ret41* carried a mutation in DET2 (de-etiolated-2, an enzyme catalyzing the reduction of campesterol to campastanol in the BR biosynthetic pathway). Etiolated seedlings of both mutants exhibited short hypocotyls and roots even when the *eto1* mutation was removed, indicating that the hypocotyl phenotype did not entirely depend on elevated levels of ethylene. Furthermore, addition of chemical inhibitors of ethylene biosynthesis and perception did not effectively suppress the triple response in *cesa6^ret8^* and *det2^ret41^* mutants. This indicates that the short hypocotyls in etiolated *cesa6^ret8^* and *det2^ret41^* mutants were probably caused by loss-of-function mutations of CESA6 and DET2, respectively, which both play an independent role in seedling development. However, an abundance of ethylene in eto1 enhanced the short hypocotyl phenotype in *cesa6* and *det2*. Additional experiments with EBL treatment of *eto1*, *det2-1*, and *det2^ret41^* showed that the balance between levels of ethylene and BRs was important for proper regulation of hypocotyl growth [13].

Not only cell elongation, but also gravitropic growth is also crucial for proper shoot development. Vandenbussche et al. [14] have shown that ethylene and BRs have opposing effects in regulating shoot gravitropism in darkness—ethylene enhances and BRs reduce gravitropic growth. Experiments in the presence of ethylene inhibitors showed that a lack of ethylene signaling enhances BR sensitivity, suggesting that endogenous ethylene may stimulate shoot gravitropism by reducing the sensitivity to BRs. It is probable that ethylene and BRs control the same downstream components even though they act in opposite ways. Additional analysis showed that both hormones regulate overlapping sets of *AUX*/*IAA* genes, implying that the effect of both hormones is performed through auxin signaling [14].

**Figure 2:**
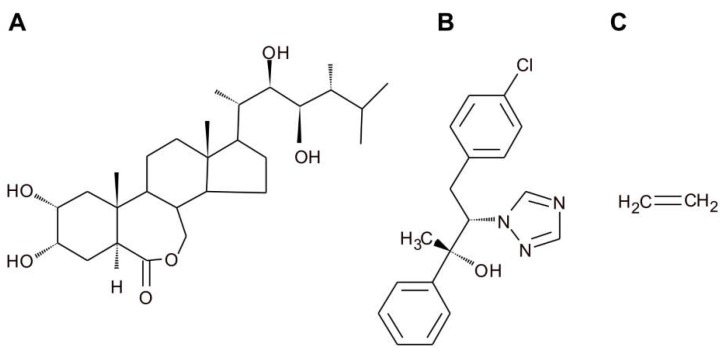
Structures of 24-epibrassinolide (**A**); brassinazole (**B**); and ethylene (**C**).

Early development of the *Arabidopsis* hypocotyl is accompanied by the formation of an apical hook, which protects the shoot apical meristem cotyledons as the seedling grows through the soil. As apical hook development is an important process following seed germination, all phases of hook development, such as hook formation, hook maintenance, and opening of the hook, are tightly regulated by the complex crosstalk of multiple hormones [15]. Both BRs and ethylene have been demonstrated to be indispensable for hook development [16]. Experiments have indicated that ethylene prolongs the formation phase of the hook development, whereas BRs prolong the maintenance phase, thereby delaying the hook opening phase [16]. Moreover, additional observations of the hook development process in plants treated with ethylene precursor ACC, EBL, (Figure 2) and an inhibitor of BR biosynthesis, brassinazole (BRZ, Figure 2), showed that ethylene-induced exaggeration of the apical hook curvature and shortening of the maintenance phase require normal BR biosynthesis. These findings were confirmed by various experiments investigating diverse BR biosynthesis as well as ethylene signaling mutants.

## 4. Flowering

The formation of flowers is a critical developmental stage because it has a direct influence on plant reproduction and yield. The *Cucurbitaceae* family is well-known for its diversity of sex expression phenotypes. Generally, in these plants, male flowers are produced early during plant development, followed later by female or bisexual flower production. Popadopoulou and Grumet [17] investigated whether BRs are involved in this process of cucurbit sex expression. They chose three different species (cucumber, melon, and zucchini) as experimental model plants. After treatment of cucumber plants with BRs, a shorter duration before appearance of the first female flower and increased production of female buds were observed. At the same time, ethylene production rose, suggesting that the effect of BRs was mediated by ethylene. Although zucchini and melon plants showed a similar increase in ethylene production as cucumber, increased femaleness was not observed in these plants after treatment with BRs. It was deduced that this was possibly because different species have different sensitivity to ethylene. Thus, in the proposed mechanism of interplay between BRs and ethylene during flower development in *Cucurbitaceae*, BRs were assumed to act indirectly via increased ethylene production with an increase in femaleness dependent on the sensitivity of specific species to ethylene.

Similar results demonstrating that ethylene has a major effect on sexual expression and flower development were shown in a more recent study from 2011. Manzano and co-workers [18] studied the effect of ethylene and BRs on flower development in different lines of *Cucurbita pepo* plants, i.e., *Bolognese (Bog)* and *Vegetable Spaghetti (Veg)*, which differ in ethylene production and sensitivity. The results showed variation in the sensitivity to ethylene among the analyzed genotypes. In the *Veg* line, ethephon (ethylene-releasing compound) induced earlier and higher production of female flowers, whereas in the *Bog* line, treatment with ethephon did not significantly alter the sexual expression. Additional data showed that the *Bog* line produced more ethylene and was more sensitive to this hormone, whereas the *Veg* line was characterized by lower production of and less sensitivity to ethylene, suggesting that this was the reason why both lines differed in their sexual expression. Further results indicated that BRs play a minor role in the control of sexual expression in *Cucurbita pepo* in comparison with ethylene [18]. The authors suggested that BR-induced ethylene may be dependent on the ethylene response, since treatment with BRZ (an inhibitor of BR biosynthesis) reduced ethylene production in the *Bog* line but increased it in the ethylene insensitive *Veg* line. Taken together, these results agreed with the previous study that the differential effect of BRs on the sexual expression of the different genotypes of *Cucurbita pepo* is probably due to the different sensitivity of these lines to ethylene [18].

## 5. Ripening and Postharvest Development of Fruit

The terminal stage of plant development is ripening of fruit, which makes fruit attractive and palatable to many seed-dispersing organisms. Because ripe fruit also represents a large proportion of the human diet, ripening makes fruit a valuable agricultural commodity. The process of ripening includes biochemical and physiological changes, such as modification of cell wall structure, conversion of starch to sugars, alterations in pigment biosynthesis, and heightened levels of flavor and aromatic volatiles. Based on respiration and ethylene biosynthesis rates, two major classifications of ripening fruit can be distinguished, i.e., climacteric and non-climacteric. Ripening of climacteric fruits, such as tomatoes, cucurbits, avocados, and bananas, is accompanied by increased respiration and ethylene biosynthesis. In contrast, non-climacteric fruits, such as citrus, do not require ethylene for their ripening [19].

The effect of BRs on quality attributes of ripening fruits and ethylene synthesis has been investigated in a recent study [20]. Tomatoes, typical climacteric fruits, were used as a model system for studying the role of BRs and ethylene during ripening. Changes in gene expression of BR synthesis were observed during tomato fruit development, suggesting that BRs might play an important role in this process This was confirmed by other experiments, in which BL-treated tomato fruits showed decreased total chlorophyll content and increased lycopene content, whereas fruits treated with BRZ displayed minor degradation of chlorophyll and lower lycopene content than the control or BR treated tomatoes. Overall, BL treatment accelerated ripening of tomato fruit, whereas treatment with BRZ delayed ripening. The same study showed that BRs can accelerate postharvest ripening of tomatoes, probably via increased ethylene production. This was demonstrated by gene expression analysis, which showed a sharp increase in the expression of genes involved in the regulation of ACS and ACO protein synthesis (LeACS2, LeASC4, LeACO1, and LeACO4) in BR treated fruit. In contrast, transcript levels of these genes were significantly depressed in tomatoes treated with BRZ [20].

A very recent study dealing with roles of BRs and ethylene during the fruit ripening point out, that at least in case of bananas, endogenous and exogenous BRs can play opposite roles in the process of ripening. In this work, authors proved that application of different concentrations of BRs promote the ripening of bananas, possibly via up-regulation of ethylene biosynthetic genes and consequently the acceleration of ethylene production. Furthermore the authors characterized three *BZR* genes in bananas (*MaBZR1, MaBZR2, and MaBZR3*). These genes encode proteins (MaBZR1-3) which belong to BZR1/BES1 transcription factors family with a central role in BR response. Both the continuous decrease of *MaBZR1-MaBZR3* expression in process of ripening as well as the suppression of a *MaBZR1-3* promoter activity indicate that MaBZR1-MaBZR3 play negative role in banana ripening. In addition, MaBZR1/2 act like a transcription inhibitors with a binding activity to element present in the promoters of ethylene biosynthetic genes (*MaASC1, MaACO13, and MaACO14*). For better understanding how high levels of BRs affect the BZR1/BES1 module regulating ethylene biosynthetic genes that turn in increase ethylene production more research is still needed [21].

Another recent article [22] focused on non-climacteric fruits and the effect of BRs/ethylene on their ripening. In this work, strawberries were used as a model study of non-climacteric fruits and they were treated with an exogenous spray of ethylene (ethephon) and EBL. The results showed that the level of phenolic compounds was influenced by both phytohormones: application of BRs tended to reduce the phenolic compound content, whereas ethylene treatment increased it. High levels of phenolic compounds caused by ethylene treatment resulted in senescence, whereas reduction of the phenolic content by BRs promoted fruit conservation as a result of increased antioxidant activity.

## 6. Stress Response

**Figure 3:**
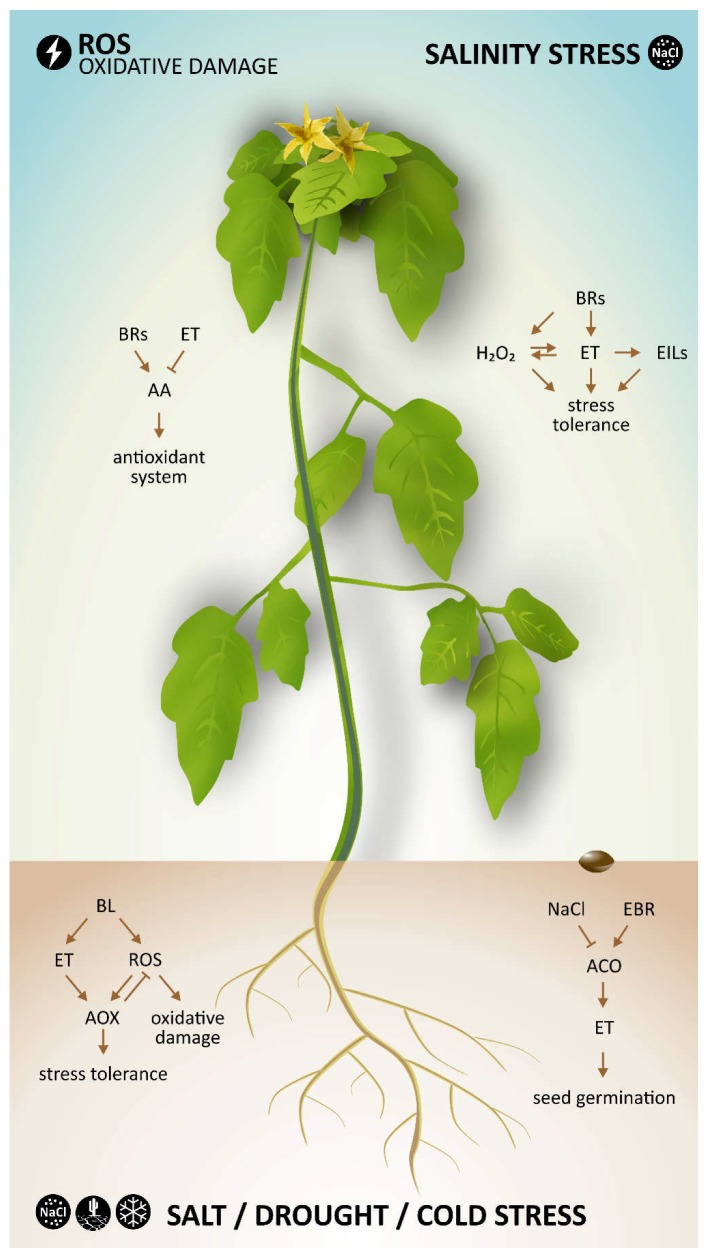
A general simplify model of BRs and ethylene interaction during abiotic stresses. Arrows indicate stimulatory effect and blunted lines indicate inhibitory effect. See corresponding sections of the text for details and references.

Both hormones (BRs and ethylene) not only play a role in plant growth and development but are also well known as hormones involved in plant responses to biotic and abiotic stresses [23,24,25,26]. The main interactions between BRs and ethylene during various abiotic stress conditions are presented in Table 1 and Figure 3.

One example by which these two hormones interact during abiotic stresses is by inducing an alternative respiratory pathway, as suggested in a recent work [27]. In this study, cucumber seedlings were exposed to salt, drought, and cold stress conditions. Pretreatment with BL (the most active BR) resulted in enhanced ethylene biosynthesis and capacity of the alternative oxidase pathway (AOX) in cucumber seedlings under stress conditions. After additional experiments investigating the relationship between ethylene and ROS (H_2_O_2_), a hypothetical model describing the function of BL, ethylene and ROS in the BL-induced AOX capacity was proposed. In this model, BRs induced ethylene and ROS generation, which subsequently enhanced AOX capacity. Enhanced activity of AOX can eliminate excess ROS generation to avoid oxidative damage in plant cells and improve their stress tolerance.

The rate of transpiration and plant water loss is regulated by the opening and closing of stomata, microscopic pores on the surfaces of leaves and stems which are bounded by two guard cells. Hence, stomata play a key role in a plant’s protection against water stress and pathogens. Stomatal opening and closing relies on reversible fluctuations of turgor and osmotically induced water flow in the guard cells. This crucial movement is triggered by various endogenous and exogenous stimuli. Thus, investigation of this opening/closing mechanism is important for understanding how plants defend against water stress and pathogens [28]. Stomatal movement is regulated by multiple plant hormones participating in a complex network of signaling pathways. The best known plant hormone linked with stomatal closure is ABA (abscisic acid), but a recent study has shown that BRs and ethylene also influence this process [29]. The results also indicated that BRs close stomata in a dose- and time-dependent manner. Experiments with *bri1-301* mutant plants containing a mutation in the BRI1 kinase domain, which leads to reduced sensitivity to BR, showed that BRs have a specific effect on stomata closure and that functional BRI1 receptor is essential for this process. Because treatment with EBR was found to significantly increase ethylene production, the study also tested whether ethylene was involved in BR-induced stomatal closure. In further experiments, EBR-induced stomatal closure was shown to be completely abolished in Arabidopsis ethylene-insensitive mutants (*etr1-1* and *etr1-3*), suggesting that ethylene plays an essential role in mediation of BR-induced stomatal closure. Furthermore, it was shown that both H_2_O_2_ (a form of ROS) and NO (nitric oxide) are involved as signaling molecules in BR-induced stomatal closure and that the BRI1 receptor is required for generation of H_2_O_2_ and NO induced by BRs in guard cells in *Arabidopsis*. Additional experiments with ethylene synthesis and ethylene perception inhibitors, as well as with mutants exhibiting a lesion in producing H_2_O_2_ and NO, suggested that ethylene mediates BR-induced stomatal closure by inducing the synthesis of H_2_O_2_ and NO in guard cells. Finally, the study presented genetic evidence that Gα (G protein α-subunit) acts as a positive regulator and mediates the action of ethylene in BR-induced stomatal closure upstream of H_2_O_2_ and NO production. Moreover, it indicated that H_2_O_2_ induces production of NO in BR-induced stomatal closure. Based on all these findings, a model of BR-induced stomatal closure was proposed, whereby binding of BR into the BRI1 receptor induces ACS expression and ethylene synthesis. Additionally, increased ethylene activates Gα, which stimulates production of H_2_O_2_ and subsequent production of NO, culminating in stomata closure [29].

One of the most crucial processes, seed germination, is affected by various stress conditions. For instance, salinity stress suppresses seed germination. Wang et al. [30] studied the effect of BRs and ethylene on the germination of cucumber seeds under salinity stress and showed that the inhibitory effect of salt (due to the presence of NaCl) on seed germination was significantly ameliorated by addition of EBR or ACC into the incubation medium. Moreover, seed germination was greater in the presence of EBR and ACC together, suggesting that these hormones may have combined alleviating effects on seed germination under salinity stress. Changes in ethylene production were also observed in this work. In the presence of NaCl in the incubation medium, imbibed seeds produced less ethylene. Addition of EBR to the medium significantly alleviated the salt-induced suppression of ethylene production of imbibed seeds. It was also shown that the suppression of ethylene production under salt stress was caused by the inhibitory effect of NaCl on the ethylene biosynthetic enzyme ACO (ACC oxidase) and that ACO activity could be reversed by treatment with EBR. Based on these findings, it was concluded that EBR affects seed germination under saline stress conditions by regulating ethylene production via recovery of NaCl-induced suppression of ACO activity [30].

An important study dealing with salt stress and crosstalk between BRs and ethylene was recently published by Zhu et al. [31]. In this work, the mechanism by which BRs induce salt tolerance in tomato plants was investigated. An increase in H_2_O_2_ and ethylene production in tomato seedlings treated with BL was observed, indicating that H_2_O_2_ and ethylene are involved in BR-induced stress tolerance. The results also demonstrated that both BRs and ethylene could promote H_2_O_2_ generation. Based on the results, a model for interactions between BRs, ethylene and ROS during salt stress was proposed. The model considered that BRs affect ethylene biosynthesis and signaling by increasing ACS (ethylene synthesis hormone) activity and stabilizing EILs (ethylene-insensitive3-like, ethylene transcription factor family), respectively, which is at least partially caused by BR-induced generation of H_2_O_2_. Further, increased levels of both ethylene and H_2_O_2_ lead to salt stress tolerance [31].

In plants, many stress conditions can cause oxidative damage. Thus, plant cells need a sophisticated central antioxidant system. Interaction of ascorbic acid (AA) and glutathione (GSH) play a crucial role in this antioxidant system to protect plants against oxidative damage. Ascorbic acid also has other physiological roles, e.g., regulation of photosynthesis and cell growth in plants [32]. Both BRs and ethylene have been shown to alter ascorbic acid-glutathione (AA-GSH) levels in tomato plants. Using a combination of genetics and chemical application, Mazorra et al. [33] showed that BRs and ethylene signaling pathways act antagonistically during regulation of AA content in tomato leaves, i.e., BRs promote AA accumulation in tomato leaves, whereas ethylene suppresses it. However, this antagonistic regulation of AA content seems to occur via independent mechanisms, i.e., normal ethylene signaling is not required for the BR effect and endogenous BRs are not critical for ethylene action [33].

Another study into the BR-ethylene interplay during protective processes against salt stress used DI-31, a BR analogue with a spiroketalic ring instead of the typical BR side chain as a cheaper alternative of BR. Lettuce plants were chosen as an experimental model of a moderately salt tolerant vegetable. After saline treatment (100 mM NaCl), a decrease in the fresh weight of both roots and shoots was observed. Pretreatment with DI-31 decreased the negative effect of salinity on the fresh weight and prevented the reduction in weight of lettuce plants. The effect of this BR analogue on ethylene emission was also examined. Without pretreatment with DI-31, plants produced more ethylene, whereas treatment with DI-31 caused a decrease in ethylene synthesis. A high correlation between the fresh weight and ethylene level caused by salt stress and possible DI-31 treatment was also observed, indicating that the synthesis of ethylene and reduction of plant weight were the result of salinity stress and that BR treatment enabled better tolerance to salinity. In addition, free ACC levels highly correlated with ethylene emission caused by NaCl treatment, which may signify that activation of ACO and ACS activity due to NaCl. DI-31 pretreatment decreased the free ACC content in tested lettuce plants. It was suggested that this BR analogue may cause lower activity of ethylene biosynthetic enzymes, e.g., ACC synthase or ACC oxidase, thus decreasing ACC and ethylene production during salinity stress and helping to protect lettuce plants against salinity [34].

Various abiotic stress conditions, such as drought or salinity, also influence symbioses between plants and microorganisms, and subsequently the uptake of essential nutrients. A recently identified ethylene signaling mutant of pea *Psein2* [35] has been studied to examine whether the interaction between BRs and ethylene may influence mycorrhizal development. Compared with wild-type pea plants, *Psein2* mutants exhibited a significant increase in the number of nodules formed for a given root mass. Moreover, these nodules were smaller and more closely spaced. After treatment with ethephon, an ethylene-releasing compound, elevated ethylene levels, which can occur in plants under stress, were achieved. In wild-type plants, ethephon treatment caused a significant reduction of fungal colonization of roots, whereas this response was absent in *ein2* mutants. These results suggest that ethylene is a negative regulator of mycorrhizal colonization. A reduced number of nodules was also characteristic for the brassinosteroid-deficient mutant *lk* [36]. To examine the interaction between BRs and ethylene on nodulation, phenotypes of the double mutant *lk ein2* were examined. Compared to *lk* single mutants, *ein2* background dwarf *lk* mutants showed considerably increased numbers of nodules and reduced nodule spacing. Nodules on the double mutant were found to be pink and appeared functional. These data suggest that BRs may stimulate initiation of nodules by affecting ethylene levels but do not affect following nodule development. With regard to arbuscular mycorrhizas, the *lk* mutation was found to reduce total root colonization by the fungus. Using the *lk ein2* double mutant, interaction of BRs and ethylene during this process could be tested. It was observed that the decrease in mycorrhizal colonization in *lk* mutant plants was comparable with low arbuscular colonization of *lk ein2* mutants, indicating that BRs have a primary effect on mycorrhizal colonization rather than acting indirectly via altered ethylene production. In summary, by using genetic studies, it was shown that ethylene influences both nodule number and arbuscular mycorrhizal colonization. However, further experiments with *lk ein2* double mutants suggested that a major part of the BR effect on modulation number may be due to elevated ethylene production, whereas the effect of BRs on colonization by mycorrhizal fungi is likely direct rather than indirect via ethylene signaling [37]. Another example of using symbiosis to overcome biotic and abiotic stresses is a microbial association of endophytic bacterium (*Enterobacter* sp. SA 187) and the desert pioneer plant *Indigofera argentea* Burm.f. (*Fabaceae*) [38]. Following experiments have shown that *Enterobacter* sp. SA 187 enhances yield of important crop plant—alfalfa (*Medicago sativa* L.) and also growth of *Arabidopsis thaliana*. Together with an ability to induce salt stress tolerance in *Arabidopsis*, *Enterobacter* sp. SA 187 has a high potential as a biological solution for improving crop production [39]. In Zélicourt et al. [39] authors further revealed that this induction of salt stress tolerance is caused via production of bacterial 2-keto-4-methylthiobutyric acid (KMBA) which is known for its conversion into ethylene, corresponding with opinion that ethylene plays positive roles in salinity response. However few studies present that some mutant plants with knock-out mutation in ACSs (crucial enzymes in ethylene biosynthesis) show increased salinity-tolerance. This discrepancy and more about the role of ethylene in plants during salinity stress is summarized in a recent review [40].

Rice is the most important basic food crop for the world’s population, so in recent years, there have been intensively explored mechanisms to increase the resistance of this plant to stress. Kumar and co-authors [41] characterized a gene *OsSta2* whose overexpression increases a tolerance of rice plants to oxidative and salt stresses. Because plants with the overexpressed *OsSta2* also showed increased responsiveness to exogenous abscisic acid (ABA), authors suggest that this gene plays a role in the ABA signaling pathway during the stress response [41]. This observation could help to complete a proposed model of ABA-dependent gene regulation mediated by OsPYL/RCAR5 in rice proposed by Kim et al. [42]. Further research dealing with stress tolerance of rice plants has revealed the importance of dehydrin gene *OsDhn1*. Rice plants overexpressing this gene show higher tolerance to drought and salt stress. This advantage is probably caused by ROS scavenging and reducing the oxidative damage [43].

For better understanding of stress response mechanism and facilitating molecular breeding it is very important to provide the genome-wide studies identifying gene families with important roles in stress responses. These studies are for example recently providing in the *Cucurbitaceae* species, where AP2/EREBP (APETALA2/ethylene responsive element binding protein) one of the largest gene families were identified and classified [44]. These genes play important roles in dealing with various environmental stresses. Further genome-wide identification provided in the *Cucurbitaceae* species was focused on the dehydrin genes encoding dehydrines—hydrophilic proteins act like molecular chaperons playing crucial role in the process of abiotic stress tolerance [45]. Both of these studies could be essential for future breeding of new *Cucurbitaceae* cultivars with stress tolerance.

Mentioning transcription factors involved in plant responses to various stresses, a novel orthologue (*MsERF11*) of ethylene response factor gene has been isolated from alfalfa (*Medicago sativa* L.), this gene encodes a nuclear located protein which as a transcription factor plays important roles during biotic or abiotic stress conditions. Because in additional experiments transgenic *Arabidopsis* plants with transferred *MsERF11* gene showed enhanced tolerances to salt stress, Chen et al. propose the potential of MsERF11 in agriculture for improving crop’s salt tolerance [46].

## 7. Summary

To summarize, the interplay between BRs and ethylene plays an important role during all developmental phases of the plant life cycle, as well as during biotic and abiotic stresses. In this article, we reviewed the synergistic effect of these hormones on root growth, seed germination under salt stress, stomatal closure, fruit ripening, and sex expression in the *Cucurbitaceae* family. The antagonistic effect of BRs and ethylene was also discussed, namely expression of the expansine gene *AtEXPA5* in *Arabidopsis* during hypocotyl growth and gravitropic growth of hypocotyls in darkness. Clearly, phytohormonal crosstalk is a complex area in which plenty of interactions remain unknown and requires further investigation by using novel approaches such as genome-wide epigenetic analyses or next-generation transcriptome sequencing of plants after BR or ethylene treatment could help clarify the mechanism of interaction between these essential plant growth regulators. The possibly understanding of the synergistic and antagonistic cross-talks of crucial plant hormones such as brassinosteroids and ethylene give us the huge potential to improve stress tolerance and yield of important agricultural crops.

## Figures and Tables

**Table 1:** Interactions of brassinosteroids and ethylene during various abiotic stresses.

Type of Stress	Species	Applied Regulator	Hormonal Interactions	Physiological Effect	References
salt drought cold	*Cucumis sativus*	BL	BL enhanced ET biosynthesis	BRs induced ET and ROS generation, which subsequently enhanced AOX capacity leading to increase of stress tolerance	Wei et al., 2015 [27]
salt	*Cucumis sativus*	EBR	EBR ameliorated the inhibitory effect of salt on ethylene production	EBR affects seed germination under saline stress conditions by regulating ethylene production via recovery of NaCl-induced suppression of ACO activity	Wang et al., 2011 [30]
salt	*Solanum lycopersicum*	BL	BRs affect ethylene biosynthesis and signaling by increasing ACS and stabilizing EILs respectively	BRs induce generation of ET and H_2_O_2_ and increased levels of ET and H_2_O_2_ lead to salt stress tolerance	Zhu et al., 2016 [31]
oxidative	*Solanum lycopersicum*	EBL 1-MCP	BRs and ET signaling pathways act antagonistically during regulation of AA content in leaves	BRs promote AA accumulation in tomato leaves, whereas ET suppresses it.	Mazorra et al., 2014 [33]
salt	*Lactuca sativa*	DI-31	DI-31 caused a decrease in ethylene synthesis	Pretreatment with DI-31 decrease the negative effect of salinity on the fresh weight and prevent the reduction in weight of lettuce plants	Serna et al., 2015 [34]
